# Analysis of X-ray scattering microstructure data for implementation in numerical simulations of ocular biomechanical behaviour

**DOI:** 10.1371/journal.pone.0214770

**Published:** 2019-04-01

**Authors:** Dong Zhou, Ashkan Eliasy, Ahmed Abass, Petar Markov, Charles Whitford, Craig Boote, Alexander Movchan, Natalia Movchan, Ahmed Elsheikh

**Affiliations:** 1 School of Engineering, University of Liverpool, Liverpool, United Kingdom; 2 School of Optometry and Vision Sciences, Cardiff University, Cardiff, United Kingdom; 3 The Manufacturing Technology Centre, Liverpool, United Kingdom; 4 Department of Mathematical Sciences, University of Liverpool, Liverpool, United Kingdom; 5 National Institute for Health Research Biomedical Research Centre for Ophthalmology, Moorfields Eye Hospital National Health Service Foundation Trust and University College London Institute of Ophthalmology, London, United Kingdom; 6 School of Biological Science and Biomedical Engineering, Beihang University, Beijing, China; Keio University School of Medicine, JAPAN

## Abstract

This study aimed to analyse microstructure data on the density and orientation of collagen fibrils in whole eye globes and to propose an effective method for the preparation of data for use in numerical simulations of the eye’s biomechanical performance. Wide-angle X-ray scattering was applied to seven healthy ex-vivo human eyes. Each eye was dissected into an anterior and a posterior cup, and radial incisions were used to flatten the tissue before microstructure characterisation. A method was developed to use the microstructure data obtained for the dissected tissue to build realistic 3D maps of fibril density and orientation covering the whole eye globe. At the central cornea, 61.5±2.3% of fibrils were aligned within 45° sectors surrounding the two orthogonal directions. In contrast, more than one-third of the total fibril content was concentrated along the circumferential direction at the limbus (37.0±2.4%) and around the optic nerve head (34.8±2.1%). The insertion locations of the four recti muscles exhibited a preference in the meridional direction near the equator (38.6±3.9%). There was also a significant difference in fibril density between the limbus and other regions (ratio = 1.91±0.45, p <0.01 at the central cornea and ratio = 0.80±0.21, p <0.01 at the posterior pole). Characterisation of collagen fibril density and orientation across the whole ocular surface has been possible but required the use of a technique that involved tissue dissection and hence caused tissue damage. The method presented in this paper aimed to minimise the effect of dissection on the quality of obtained data and was successful in identifying fibril distribution trends that were compatible with earlier studies, which concentrated on localised areas of the ocular globe.

## Introduction

The cornea is an important optical component of the eye that contributes more than two-thirds of its optical power [1, 2]. In addition, it provides protection for the inner ocular contents and maintains a shape that is suitable for precise and stable light refraction under the dynamic intraocular pressure (IOP) [3–7]. These characteristics are mostly governed by the corneoscleral microstructure, in particular, the organisation of collagen fibrils–the main load carrying components of ocular tissue.

The stroma, where most of the collagen fibrils are situated, accounts for 90% of the cornea’s thickness and has the largest impact on its mechanical behaviour [8–10]. In the central cornea, the stroma has approximately 200 lamellae, each with uniformly spaced and sized fibrils, primarily running parallel to the cornea’s surface, but lamella interweaving has been observed in the anterior stroma and in the peripheral zone, close to the limbus [10–13]. X-ray scattering techniques have been widely used to quantify fibril arrangement (density and orientation) in corneal stroma [11, 14–16]. The results indicate that in healthy corneas, the fibrils in the central zone have two preferred orientations–the temporal-nasal (T-N) and superior-inferior (S-I) directions–with two-thirds of the fibrils commonly aligned within the 45° sectors surrounding these orientations. The fibril preferential orientation then changes to circumferential at the limbus with a transition zone in the paracentral area. The depth-dependent arrangement was also observed, being more orthogonal in the posterior stroma than in the anterior lamellae [5, 12].

On the other hand, as the scleral collagen is wider and has much more interweaving than the cornea, scleral microstructure is less regular compared to the corneal microstructure, its outer layer has thinner and narrower collagen bundles consisting of thin fibrils, while the inner layer has thicker and broader bundles [17]. Further, the arrangement of bundles in both outer and inner layers of the sclera appears to be random, irregular and complex.

The complex microstructure of the sclera and the previous lack of comprehensive information to quantify it have meant that the several constitutive models that are currently available to describe the ocular anisotropic material behaviour based on fibril distribution are primarily focused on the cornea [8, 18–23], while other attempts adopted simplified microstructure patterns in the sclera [24–26].

However, while a recent study has been able to quantify fibril distribution across the whole ocular globe [27], restrictions with the X-ray scattering technique, which necessitated dissecting and flattening the ocular tissue during the measurement process, have made analysis of the microstructure data and its implementation in numerical modelling challenging. The tissue dissection and flattening were not reversed efficiently when the eye’s 3D shape was reconstructed and as a result, gaps and overlaps appeared in the collagen fibril distribution.

This paper presents a strategy to analyse microstructure data obtained for whole eye globes while overcoming the challenges caused by the tissue preparation methods employed in earlier work. In particular, the study aims to address three main challenges; the discontinuity in the X-ray scattering data of flattened eye samples, the closure of gaps in the tissue in its 2D form, which were caused by dissection, and the mapping of tissue microstructure on the 3D geometry of the ocular globe.

## Methods

The experimental work described in this manuscript was approved by the Human Science Ethical Committee (School of Optometry and Vision Sciences, Cardiff University, UK) and the South East Wales Research Ethics Committee (Cardiff, UK). The institutional review board approved the use of all ocular tissue in this study; a waiver of consent was given for the donor eye globe. All tissue used in this study was obtained in accordance with the tenets of the Declaration of Helsinki, and local ethical rules were adhered to throughout. All experimental procedures were performed in accordance with the Declaration of Helsinki.

### Ex-vivo specimen preparation and X-ray scattering details

**Fig 1:**
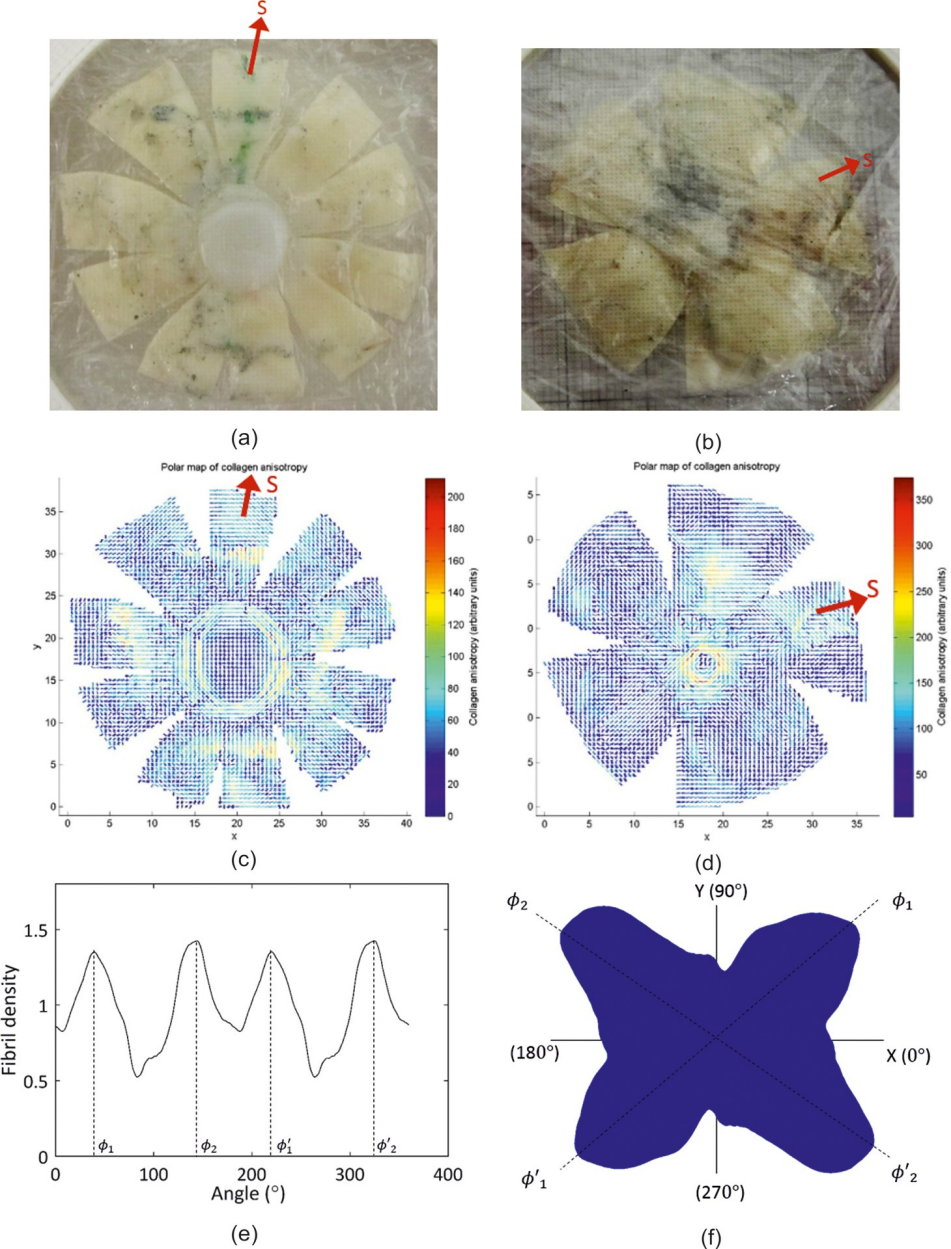
Information obtained from an eye globe of a 75 year old male donor. (a), (b) anterior and posterior parts after dissection—the red arrow marks the superior direction; (c), (d) X-ray scattering fibril orientation vector maps of anterior and posterior parts; (e) fibril density in different directions obtained at a measurement point showing four peaks in density at angles *ϕ*_1_,*ϕ*_2_,*ϕ*′_1_,*ϕ*′_2_, where *ϕ*′_1_ = *ϕ*_1_ + 180° and *ϕ*′_2_ = *ϕ*_2_ + 180°; (f) a polar plot of fibril distribution at a measurement point.

Seven ex-vivo healthy human eyes (three right eyes and four left eyes) from 7 donors aged 60–80 years were supplied by the Fondazione Banca degli Occhi del Veneto Onlus Eye Bank, Italy. They were enucleated, frozen and transported at -08°C to the University of Liverpool. The eyes were initially inflated by phosphate-buffered saline (PBS) at 17 mmHg pressure to simulate the normal intraocular pressure range in a process to counteract the effects of post-mortem swelling [13]. This was followed by fixing the tissue in 4% paraformaldehyde (PFA) then storing it at 4°C until the X-ray scanning process. Prior to data collection, the eyes were dissected along the equator as shown in Fig 1. The tissue thickness was measured at uniformly spaced points across the globe surface using a Pachette2 ultrasound Pachymeter (DGH Technology, USA).

Preparations for microstructure characterisation started with making meridional incisions to enable flattening the tissue and fixing it in a bespoke device, which was later fitted in the X-ray facility (Station I02, Diamond Light Source Synchrotron, Oxford, UK), Fig 1. Wide-angle X-ray scattering (WAXS) was performed as described previously [27]. The number of meridional incisions has been set to 10 in the anterior sclera and 5 in the posterior sclera following trials on porcine eyes in order to find a compromise between the number of incisions, which cause tissue damage and reduce data reliability, and tissue curvature, which causes problems with clamping and affects quality of measurement when the tissue is not set perpendicular to the beamline. Hemispherical clamps that would allow scanning the eye with one equatorial cut were also attempted but despite using motors with the highest resolution available commercially, the device movement between scans had unacceptably large tolerance, that was close to the 0.5 mm distance between the scanned points.

The measurements of collagen fibril microstructure were made at points with 0.5 mm spacing in two orthogonal directions. At each point, fibril content was estimated in evenly-spaced 256 orientations covering the full 360° with 0° being at at the positive direction of the cartisian X-axis, Fig 1F. This data is presented graphically in the form shown in Fig 1C and 1D [28].

The specimen preparation process led to gaps in the data collection in the locations where the tissue was incised. The flattening of the naturally curved tissue also led to geometric distortion, but this was kept at a low level by using the number of incisions shown in Fig 1. The method presented in this paper is an attempt to reverse the geometry change caused by the ocular globe being cut along the equator and incised meridionally. This has been attempted in the past, but limitations in the methodology produced overlaps and gaps in the data when transferred to 3D geometry [27]. The method further includes analysis of a large amount of raw data obtained and its preparation in a form suitable for use in finite element models of the eye.

### Gap closure

**Fig 2:**
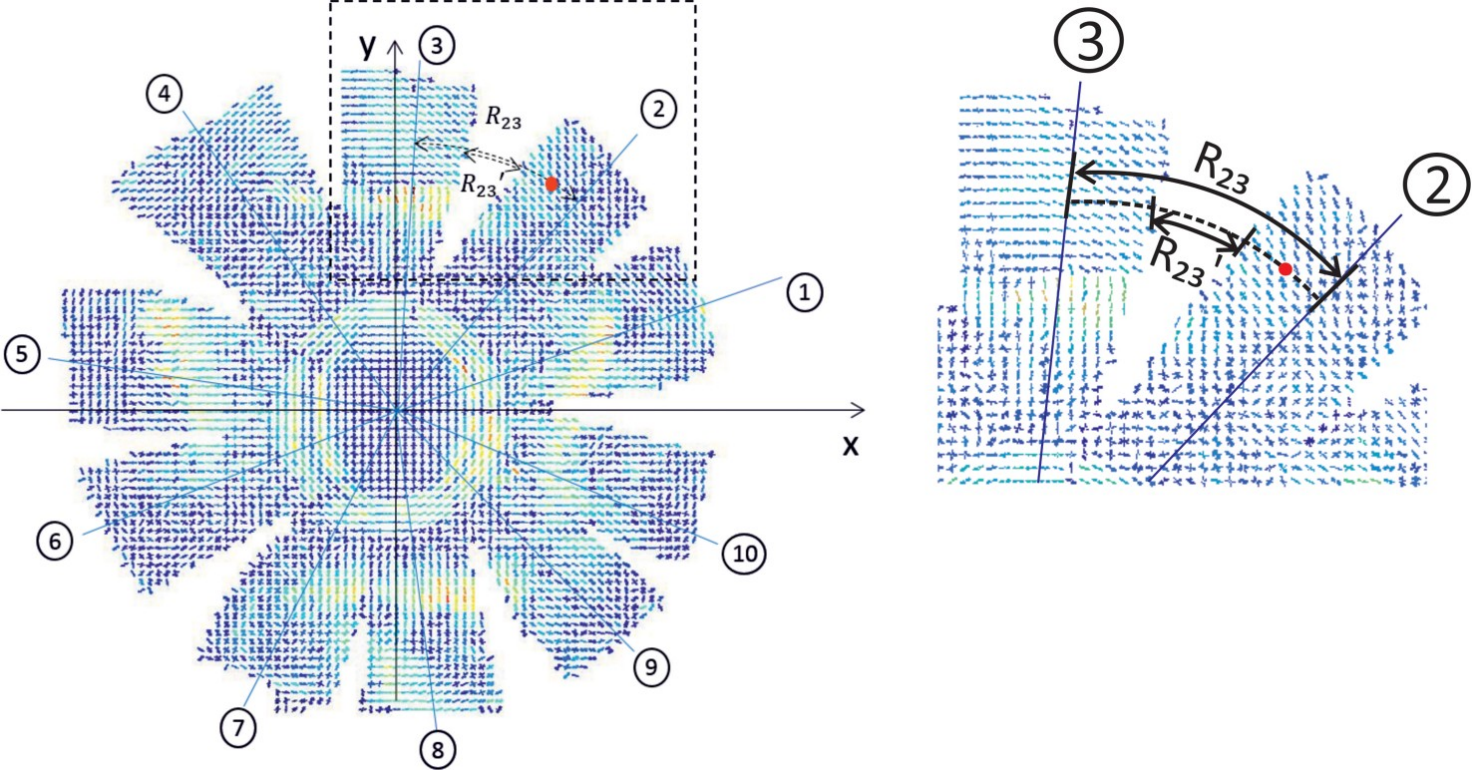
Data points on a dissected anterior segment. 1 to 8 represent the central lines located for each segment. *R*_23_ represents the arc length that passes through the red point and extends between central lines 2 and 3, while *R*_23_′ represents the part of *R*_23_ that extends between the boundaries of segments 2 and 3.

The process of closing the gaps caused by the incisions and obtaining a continuous 2D circular map for each of the anterior and posterior cups started with locating a centre point for each cup as the closest point to the geometric centre of the scanned points. From the centre point, a central straight line was located for each tissue segment, separating the scanned points included into two equal groups, Fig 2. For each scanned point (e.g. red dot), two arc lengths were calculated; one that covered the circumferential distance between the two segment centre lines that were closest to the scanned point (*R*_23_ in Fig 2), and one that quantified the gap width between the two segments for which the two centre lines were considered (*R*_23_′). With these two lengths, an expansion factor, defined as *R*_23_/(*R*_23_−*R*_23_′), was calculated and used to project the scanned point circumferentially in order to close the gaps caused by the incisions.

The next step was to fit a circle around the scanned points in their new locations while ensuring that the irregularity and roughness of edges were eliminated. Depending on how well specimens were incised, this process resulted in losing a maximum of 1.0 mm of data around the equator–between the anterior and posterior maps. The two maps were then aligned together by rotating their superior directions towards the positive Y-axis.

### Local coordinate system

At the start of the analysis process, each of the scanned points on a map had a local coordinate system that shared the same X and Y directions as the global coordinate system. Throughout the data manipulation process described above, care was taken to rotate the local coordinate system as the points were shifted circumferentially relative to the central point. As the points were fitted on a 3D surface representing the globe’s surface, the X and Y axes were oriented in the circumferential and meridional directions, respectively, with reference to the corneal apex and posterior pole for data points in the anterior and posterior hemispheres.

### Fibril trend analysis

Following gap closure and rotation of fibril distribution data at the X-ray measurement points, any preference identified in fibril alignment was recorded and compared to trends reported in earlier studies [29]. Special attention was also given to the proportion of fibrils within the 45° sectors surrounding the vertical and horizontal directions in the cornea, the circumferential direction in the limbus and around the optic nerve head, and the attachment locations of the extraocular muscles, Fig 2.

A previous study showed the mirror symmetry between the microstructure maps of left and right corneas [16]. An exercise was carried out in this study to assess whether mirror symmetry exists within the posterior part of right and left scleras. Within a circular area with 6 mm radius around the posterior pole, the fibril density was calculated (using Zernike polynomials) at each integration point based on the microstructure data obtained for each of the 7 scanned eyes. The standard deviation of the 7 densities at each integration point was then calculated as a percentage of the corresponding mean density. This step allowed calculation of the mean percentage standard deviation across the whole circular area. This exercise was conducted twice, once after flipping the right eye maps and once without flipping, and a comparison between two results was used to assess whether mirror symmetry existed in the posterior sclera.

### Data fitting

The fibril magnitude data obtained for each measurement point were normalised by dividing them by the local thickness value and the results were considered representative of fibril density. As the density data at each point were centrosymmetric, they were fitted to Zernike polynomials in half of the 256 orientations to enable the estimation of density at any point on the ocular surface and in each orientation. 128 sets of Zernike polynomials were fitted to the fibril density data at all measurement points and in the 128 data orientations considered. Once values of coefficients of the Zernike polynomials were determined, they were used to estimate the density in any of the 128 orientations and at any point on the ocular surface. As the 3D coordinates of a point on the ocular surface can be uniquely represented by the point’s inclination angle and azimuthal angle, the 3D coordinates could be translated into 2D coordinates, which then allowed the use of the 128 polynomial sets to estimate the fibril density at this point in each of the 128 orientations.

This process helped remove unexpected, localised peaks and troughs that could be caused by measurement noise [30]. Before using Zernike polynomials in this study, their effectiveness was compared to those of Gaussian-Hermite and Orthogonal Fourier-Mellin polynomials, and the results showed no significant differences in the error of fitted maps [31–33]. Hence Zernike polynomials were used in the study as they were more commonly used by researchers in this field. The general representation of Zernike polynomials takes the form:

$$Z_{n}^{m}(r,\theta )=\{\begin{array}{c} R_{n}^{\left|m\right|}\cos(m\theta )\;m\geq 0\\ R_{n}^{\left|m\right|}\sin(\left|m\right|\theta )\;m<0 \end{array}$$ (1)

Where $R_{n}^{\left|m\right|}(r)$ is the radial polynomial defined as $\sum_{i=0}^{\frac{n-|m|}{2}}\frac{{\left(-1\right)}^{i}(n-i)!r^{n-2i}}{i!(\frac{n+|m|}{2}-i)!(\frac{n-|m|}{2})!}$, (0 ≤ *r* ≤ 1), *n* and *m* are integers that represent the order of the polynomial and the angular frequency, respectively. By increasing the order, Zernike polynomials become more able to fit complex surfaces, but the process of fitting would become more time consuming and could lead to overfitting.

**Fig 3:**
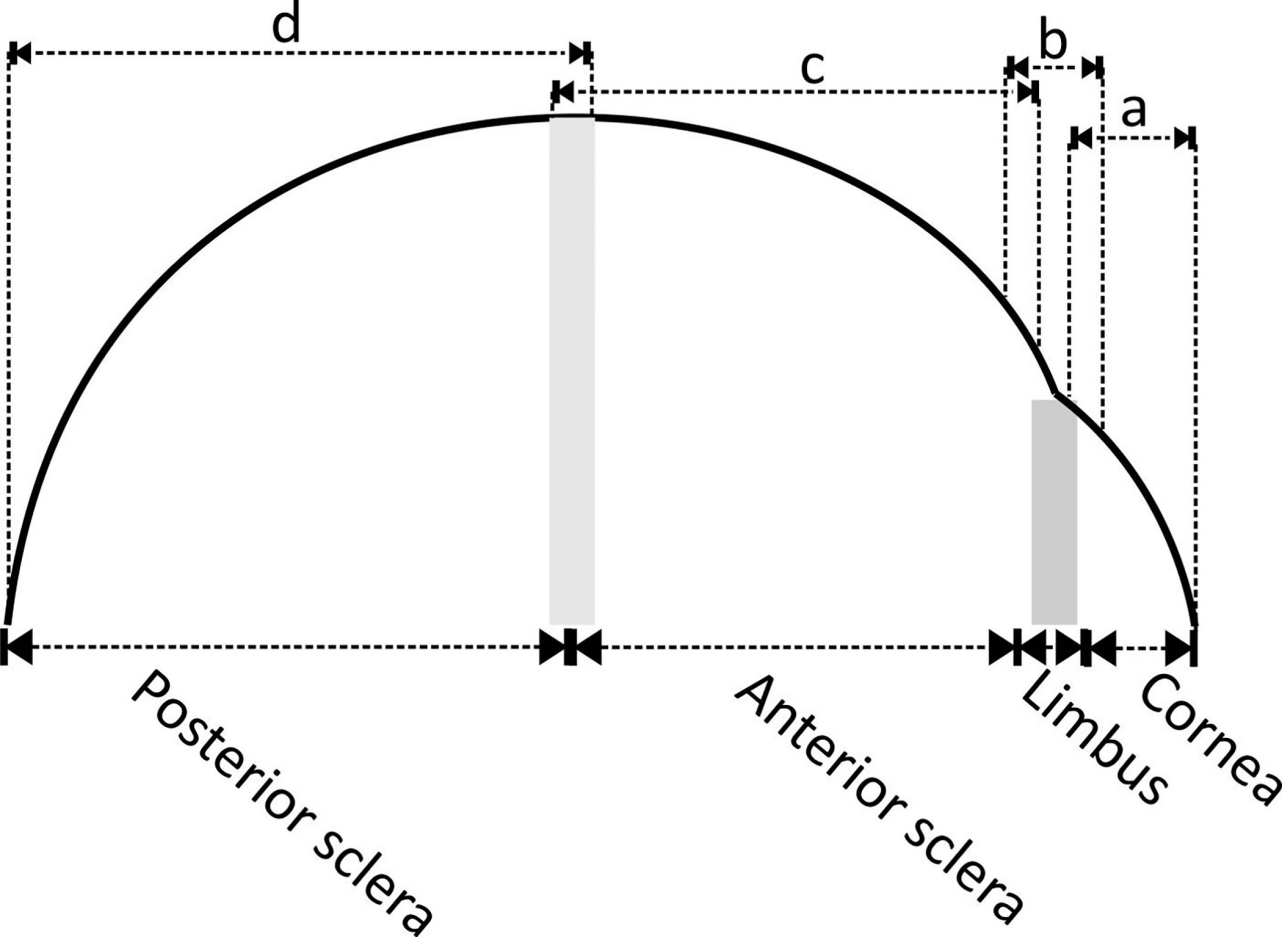
Schematic side view of an eye showing the four zones. Fibril density data were fitted to different sets of Zernike polynomials over the four zones. Overlaps were allowed between the zones to enable a smooth transition from one zone to another. Fibril density in Zones ‘a’, ‘b’, ‘c’ and ‘d’ were fitted into Zernike polynomials to enable interpolation of fibril density in the cornea, limbus, anterior sclera and posterior sclera, respectively.

Further, due to the large regional variation in fibril density, fitting all data in any of the 128 orientations to a single set of polynomials caused the loss of important features such as the distinctive orthogonal arrangement of fibrils in the central cornea. For this reason, the surface of the globe was split into 4 sections covering the cornea, limbus, anterior sclera and posterior sclera, respectively, Fig 3. A small overlap about 2.4 mm in meridional distance between every two adjacent zones was introduced to ensure a smooth transition from one zone to another.

### Order of Zernike polynomials

Comparisons were held between results obtained with Zernike polynomial orders 3 to 25. 80% of the measurement points were used in an optimisation exercise based on the least squares method to determine the polynomial coefficients, which were then used to assess the reconstruction (fitting) accuracy of the polynomials. The polynomials were subsequently used with the rest of the data (20%, ensured to have regular distribution across all ocular surface) to assess the quality of prediction and hence discover any signs of overfitting [34]. Least squares minimisation was used to calculate the polynomial coefficients, taking approximately 5 seconds to compute the coefficients’ values for a single orientation with 15 decimal places on a PC with Intel Core I7-4790 and 16GB RAM [35]. Both reconstruction and prediction errors were determined by calculating the root mean square (RMS) of differences between original and calculated values for all the measurement points in the map.

### Statistical analysis

For statistical analysis, variations in fibril density and orientation between the seven human eyes were studied. Fibril densities in different regions were compared after calculating the mean and standard deviation. Statistical significance in fibril density was assessed with the two-sample t-test using Excel (Version 2013, Microsoft) as the distribution of the measurements was found to have normality. The null hypothesis probability (p) at a 95% confidence level was calculated. The probability p is an element of the period [0,1] where values of p higher than 0.05 indicate the validity of the null hypothesis [36].

## Results

### Order of Zernike polynomials

**Fig 4:**
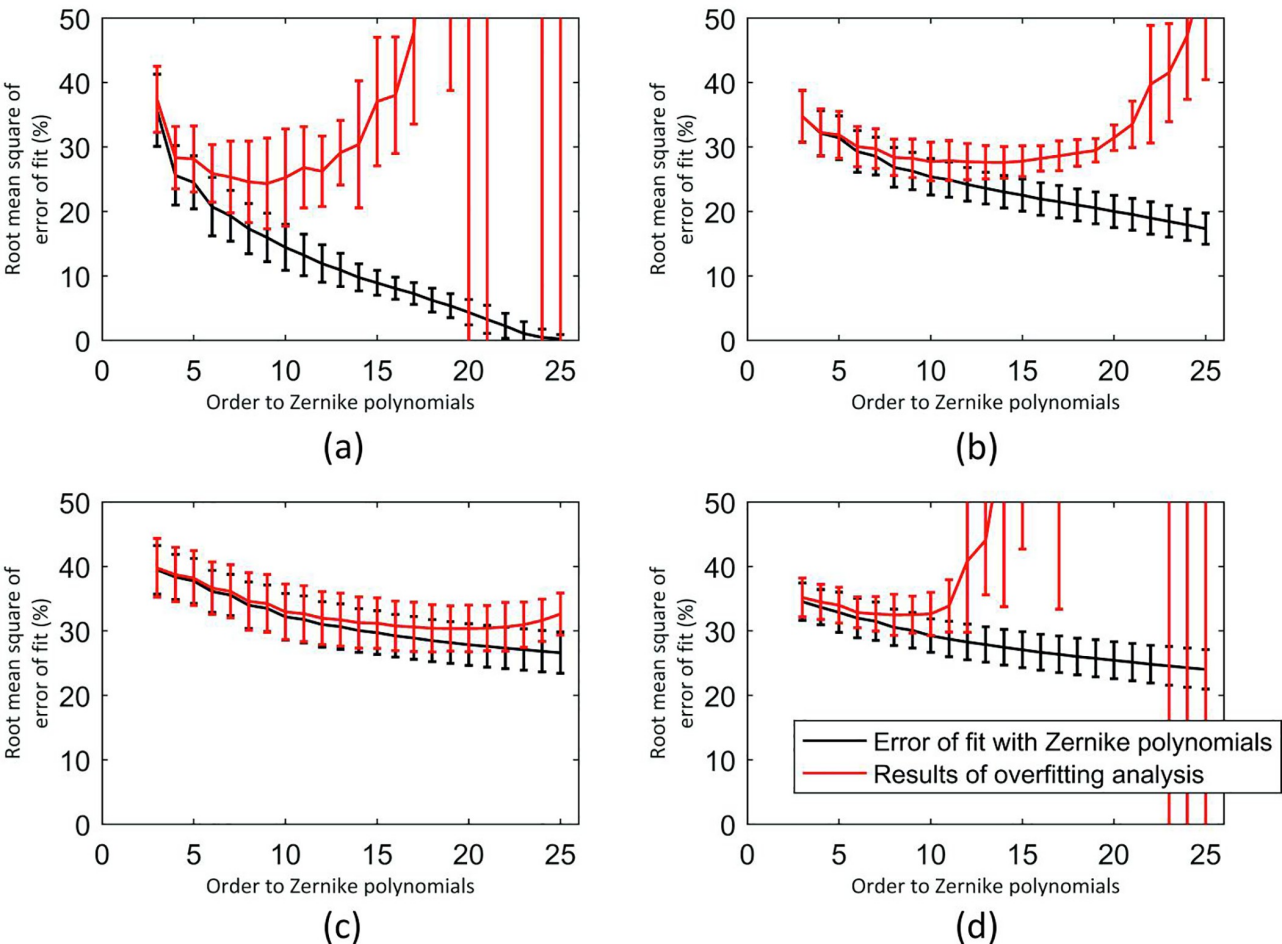
Errors of fit with Zernike polynomials, and errors caused by overfitting. (a) zone ‘a’: the cornea, (b) zone ‘b’: the limbus, (c) zone ‘c’: the anterior sclera, and (d) zone ‘d’: the posterior sclera. The separation between the red and black lines shows the overfitting effect.

The accuracy of fit of Zernike polynomials used to represent the fibril density at each of the 128 orientations, and in each of the four zones that make up the ocular surface, was estimated by the value of Root Mean Square (RMS) error of fit with the raw data, Fig 4. The reconstruction errors at all considered Zernike orders, based on 80% of the measurement data, were lower in zone ‘a’ than in other zones, possibly due to the particular regularity of fibril distribution in the cornea. However, as expected, the reconstruction errors reduced successively in all four zones with higher polynomial orders. In zone ‘a’, the RMS error reduced from 35.7±5.6% of the average value of fibril density with order 3, to nearly 0% with order 25. The corresponding reductions in other zones were from 34.8±4.0% to 17% in zone 2, from 40.4±3.8% to 26.1±3.2% in zone ‘c’, and from 36.3±2.9% to 26.6±3.1% in zone ‘d’. These reductions were also consistently observed in all 7 eyes analysed, and this has been reflected in the small standard deviation values depicted by the error bars in Fig 4.

**Fig 5:**
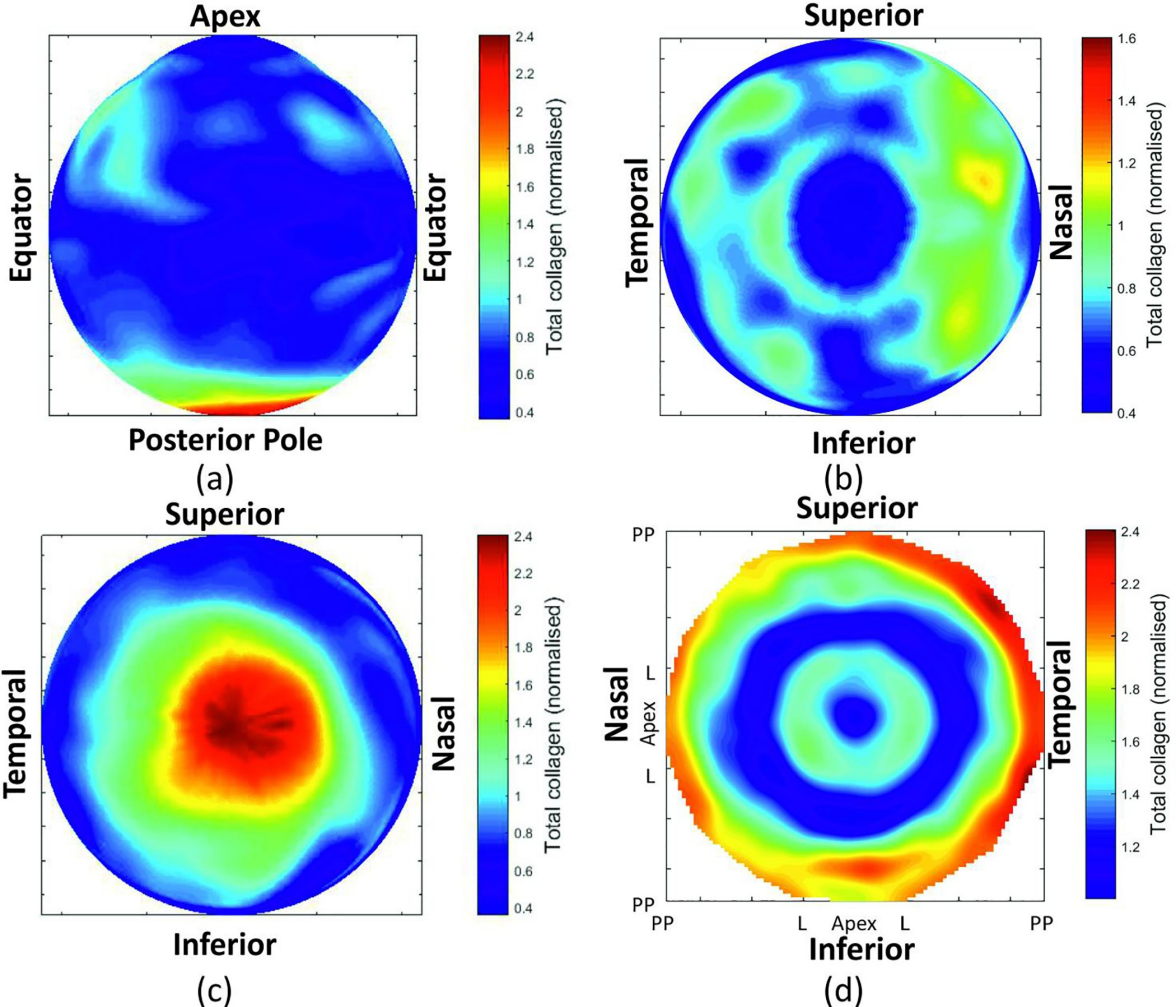
Total collagen distribution contour map. Representation of collagen distribution is shown in a 3D from (a) a front view, (b) a top view, and (c) a bottom view. Collagen distribution is also shown in (d) a 2D map with the centre representing the corneal apex and the perimeter representing the posterior pole.

Fig 4 also represents the prediction errors that referred to the remaining 20% of the data (red lines). With low order polynomials (3–7), the prediction errors were almost the same as reconstruction errors. However, with order increases, the prediction errors underwent rapid increases, indicating possible overfitting. This trend was evident in both zones ‘a’ and ‘d’, but less so in zones ‘b’ and ‘c’. A compromise was therefore made in adopting order 10 in all four zones. The results of using order 10 in a typical specimen are illustrated in Fig 5, which shows different views of the resulting fibril density map.

### Mid-line symmetry

**Fig 6:**
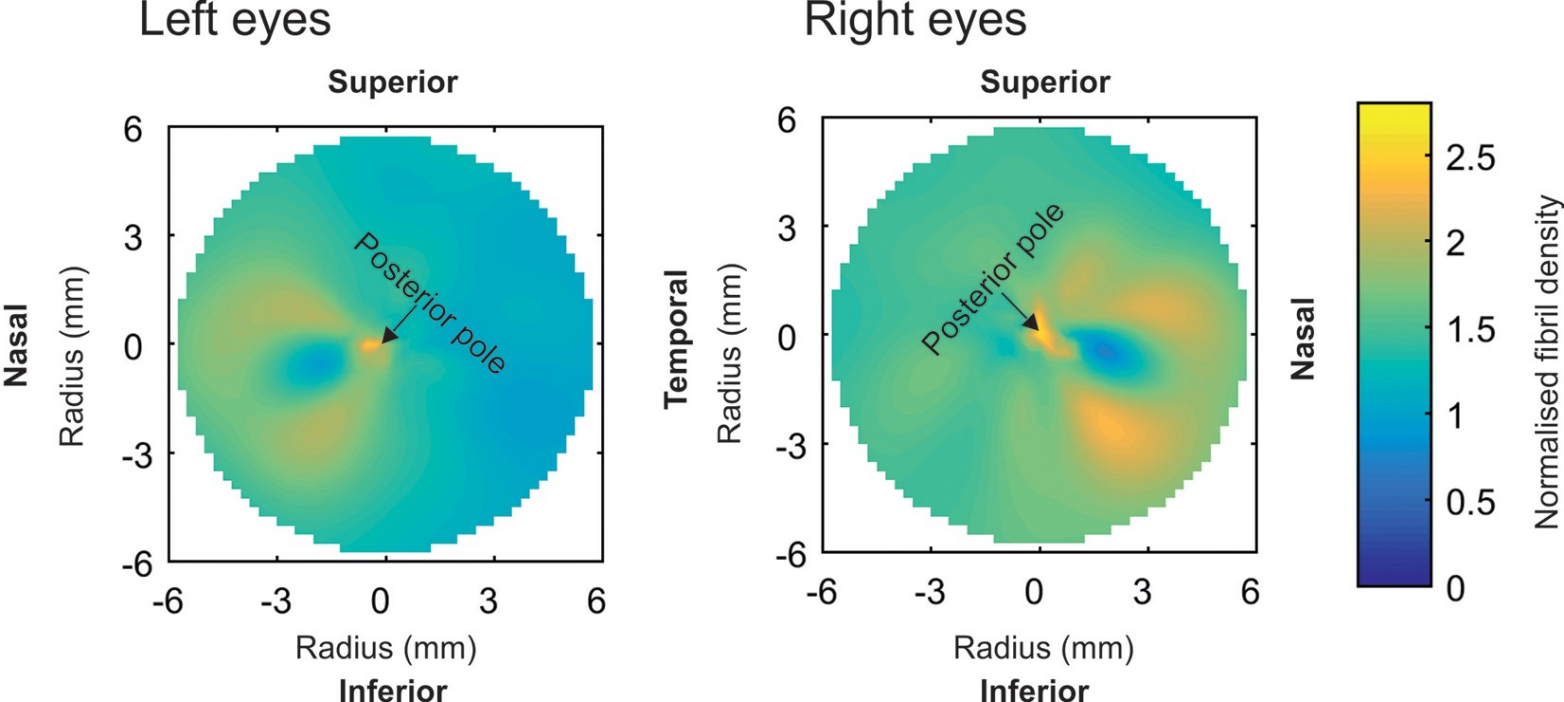
Average maps of total collagen density for the four left eyes and three right eyes.

A graphical evidence of mirror symmetry in the poterior sclera can be observed in the averaged left-eye map and right-eye map (Fig 6) especially in the area surrounding the optic nerve head. Analytically, the mean percentage SD of fibril density within the 6 mm radius area around the posterior pole was 44% after flipping the right eye maps, but increased to 57% if map flipping was not carried out. The reduction in mean SD after map flipping is an indication of the mirror symmetry of fibril distribution in the posterior sclera.

### Fibril distribution trends

**Fig 7:**
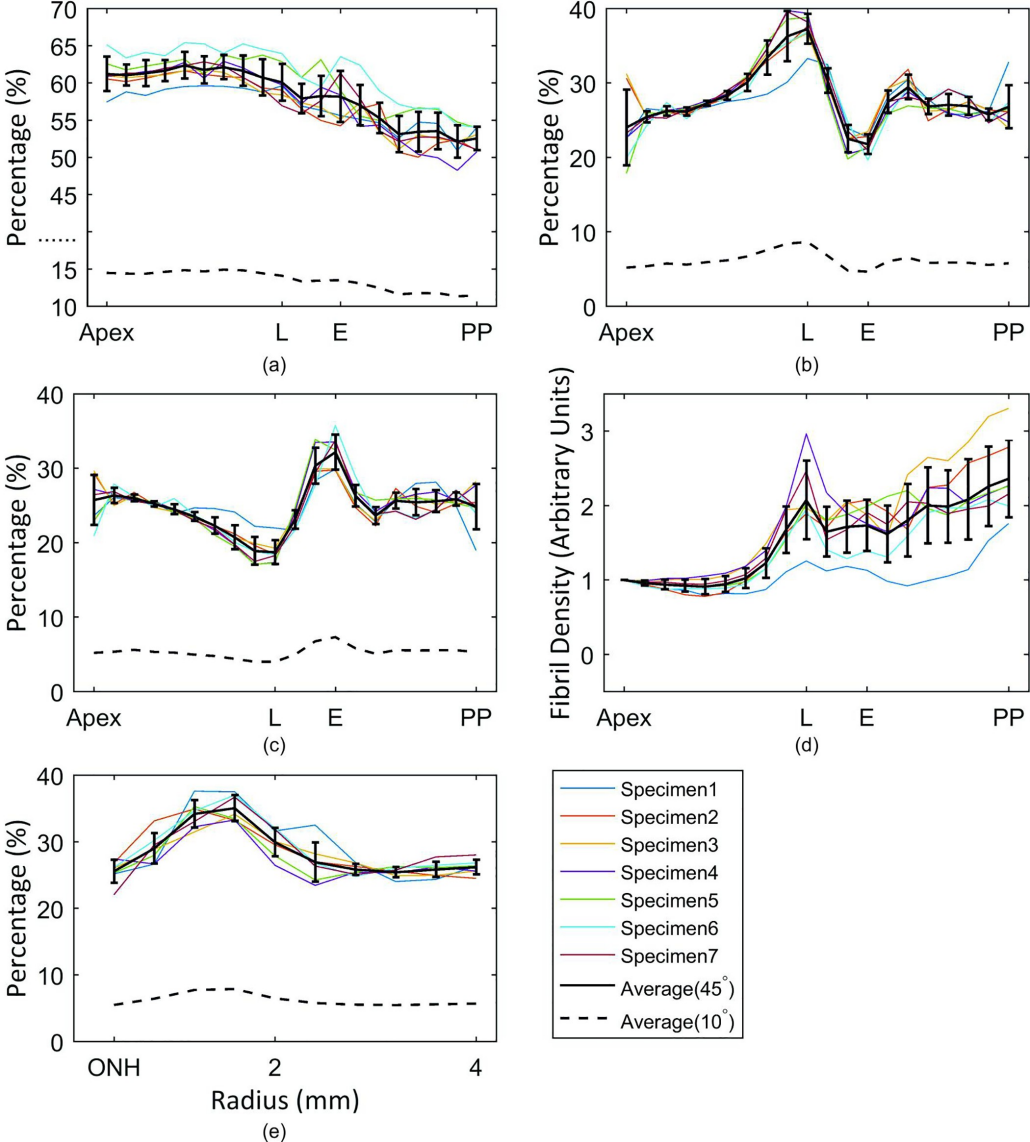
Proportion of fibrils aligned within the 45° and 10° sectors. The sectors surrounding (a) the orthogonal direction at points along the eye’s 4 main meridians, (b) the circumferential direction, (c) the meridian direction. Fibril density at all measurement points is shown relative the density at the corneal apex in (d). Fibril content in the circumferential direction around the optic nerve head (ONH) and within an area with 4 mm radius is shown in (e). Error bars represent the standard deviation of results. L = limbus; E = equator; PP = posterior pole.

Analysis of microstructure data obtained for the seven eye globes illustrated the regular arrangement of fibrils in the central cornea. 61.5±2.3% of fibrils in the central cornea (within a 3mm radius of the apex) were aligned within the 45° sectors surrounding the 4 main meridians–equally split between the temporal-nasal and superior-inferior directions. Orthogonal preferential orientation then reduced gradually away from the central cornea with the percentage within the 45° sectors becoming 52.6±1.6% at the posterior pole, Fig 7A. In contrast, the fibrils at the limbus were preferentially oriented in the circumferential direction (37.0±2.4%, Fig 7B), but this percentage decreased sharply to 22.0±1.2% near the equator. Beyond the equator, circumferential fibril density increased again to 27.4±2.4% and remained at this level until reaching the posterior pole.

The variation in circumferential distribution was reflected in, and compensated by the fibril arrangement in the meridian direction, Fig 7C. The proportion of meridional fibrils were at its minimum level (19.2±1.8%) at the limbus, before increasing to maximum values (32.2±2.1%) at the equator—at approximately the location of the extraocular muscle attachment. Beyond the equator, and up until the posterior pole, meridional fibril content was close to 25%, indicating no preferential orientation.

Total fibril density exhibited its lowest value at the central cornea but increased significantly (p <0.01) to almost twice the central values at the limbus, Fig 7D. After a sudden drop in density from the limbus to the equator, the fibril density experienced a gradual increase to a maximum value around the posterior pole, which was significantly higher than the limbus (p <0.01).

Further, a predominantly circumferential distribution was found within the 4mm-diameter zone surrounding the optic nerve head. The proportion of circumferential fibrils in this region increased from 25.7±2.1% at the centre to 34.8±2.1% at 1.75 mm radius, then decreased back to 26.2±1.0% at 4 mm radius, Fig 7E.

**Fig 8:**
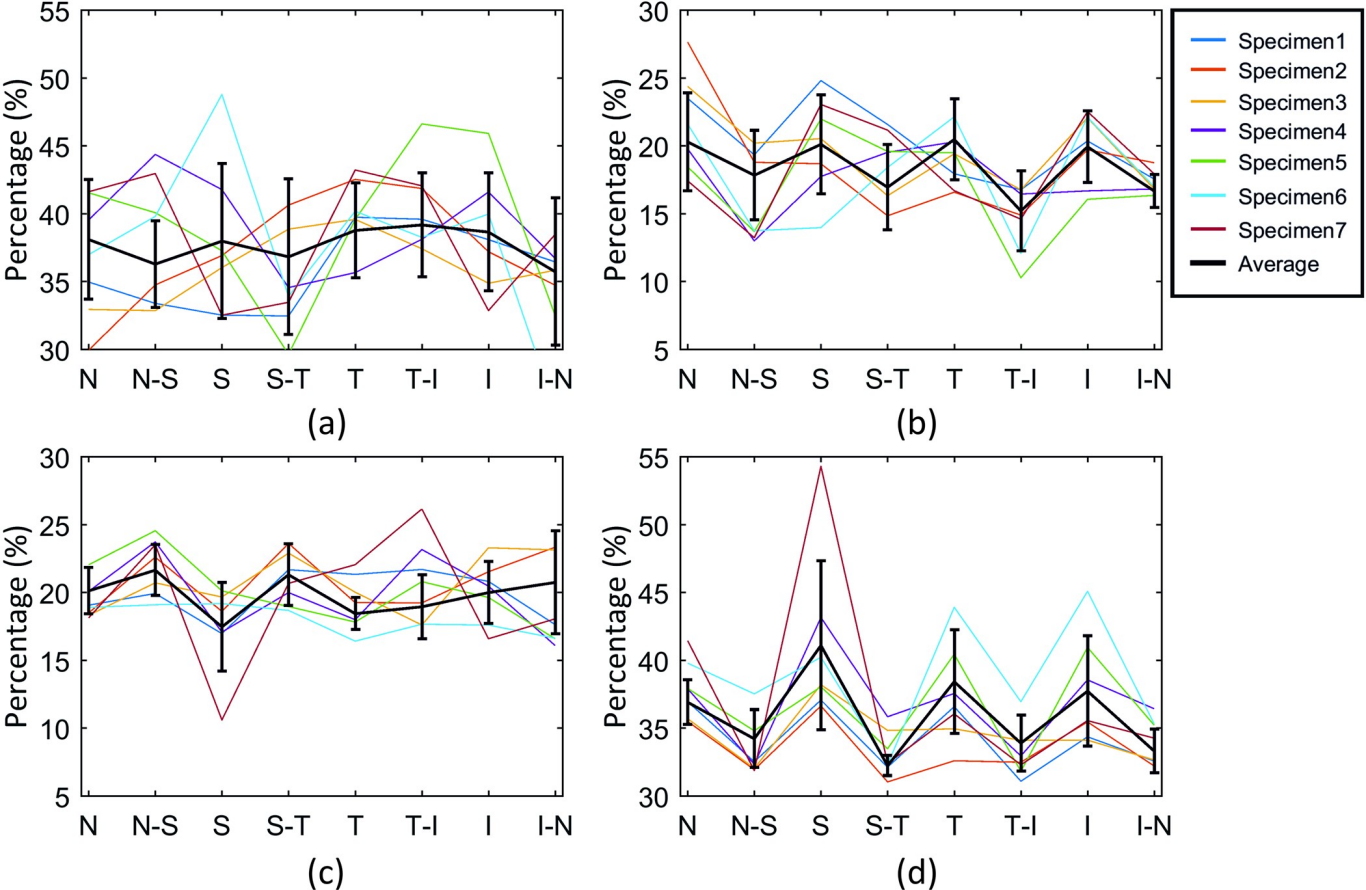
Quantification of fibril content along the eight main meridians of the eye. (N: nasal, N-S: nasal-superior, S: superior, S-T: superior-temporal, T: temporal, T-I: temporal-inferior, I: inferior, I-N: inferior-nasal). The values present the proportion of fibrils aligned within 45° sectors surrounding the (a) circumferential and (b) meridional directions at the intersection points between the limbus and the eight meridians. Also shown are equivalent values presenting the proportion of fibrils aligned within 45° sectors surrounding the (c) circumferential and (d) meridional directions at the intersection points between the equator and the eight meridians.

Further assessment of fibril distribution was carried out by considering the circumferential and meridional fibril densities along the limbus and equator, Fig 8. When fibril density along the four main meridian directions at the equatorial region was compared with fibril density along the oblique orientations, a significant difference (p< 0.01) was found. The density was higher at the cardinal meridians where the muscles are connected. The average meridian oriented fibril density (38.6±3.9%) along the four cardinal meridians was higher than the meridian oriented fibril density (33.4±1.6%) at other meridians (p< 0.01, Fig 8D). In contrast, the variation of circumferential fibril density along the limbus did not show clear trends, Fig 8A. The proportions of fibril density were about 20% in the meridional direction with the same data points in the limbus, Fig 8B. The same effect was observed for the circumferential distribution at the equator, Fig 8C.

## Discussion

This paper presents a method to translate the current X-ray scattering microstructure data covering the whole ocular globe into a form that can be utilised in building numerical models of the eye’s mechanical behaviour. Microstructure, especially the distribution of collagen fibrils, has a direct effect on the biomechanical behaviour of ocular tissue, with higher fibril content being indicative of higher mechanical stiffness and preferential orientation causing anisotropic stiffness [29, 37, 38]. Conditions, such as keratoconus and ectasia, which are known to be associated with significant reductions in ocular tissue stiffness, have also been found to be related to changes in microstructure including reduction in fibril content and loss of the regular fibril organisation commonly observed in the healthy cornea [29, 39].

This association between fibril distribution and ocular stiffness has triggered a growing body of research to build numerical models of the human eye based on its microstructure map [8, 21–23, 40, 41]. Most of this work relied on several studies conducted by the Structural Biophysics Group in Cardiff University that characterised corneal microstructure in both healthy and keratoconic eyes [11, 16, 28, 39]. Corneal microstructure anisotropy, with preferential fibril distribution in vertical and horizontal directions, was simulated mathematically as orthogonal components only [21, 41], or with the use of a dispersion factor that enables a smooth variation in direction-dependent content [8]. In parallel, an angular integration method was used to incorporate the fibril distribution data directly into a strain energy equation depicting the tissue constitutive model through a probability density function [22, 23, 40]. These significant advances made it possible to build numerical models that are representative of the distribution and orientation of fibrils, the main load carrying components of the tissue. The current study aimed to extend the scope to the full outer tunic of the ocular globe, including both the cornea and sclera, benefitting from microstructure data that for the first time covered the whole globe.

The microstructure data obtained in this study observed similar trends to those reported previously. The obtained results revealed a strong preferential orientation of fibrils in the central corneal region, with 62% of fibrils aligned within the 45^o^ sectors surrounding the temporal-nasal and superior-inferior directions–a content that is slightly lower than the 67% value reported earlier using small-angle X-ray scattering [29]. The figures also show that, on average, 37% of the fibrils have a circumferential arrangement in the limbus. This value compares to 42% previously reported for normal human corneas in an earlier study using WAXS on only two healthy corneas [42]. At the equator, fibrils were found to be preferentially aligned in the meridional direction (39% of total fibril content), and concentrated along the four cardinal meridians, at the locations where the extraocular muscles are attached to the ocular globe, Fig 1. Further, fibril distribution was found to be 35% circumferential at 1.75 mm away from the centre of the optic nerve head. This preference can be associated with the reinforcement of the peripapillary sclera, which tends to limit scleral canal expansion and tensile lamina cribrosa forces under IOP fluctuation [43, 44].

The limitations of the X-ray scattering technique have meant that the globe had to be dissected along the equator and meridionally, to enable flattening the specimens before scanning. The dissection and flattening of the anterior and posterior ocular hemispheres may have led to a relaxation of tissue and distortion in the regions immediately adjacent to the dissection. This level of interference with the globe’s 3D geometry created challenges in using the resulting microstructure data and utilising it in the construction of representative numerical models. As a limitation of the study, the X-ray scattering technique was unable to characterise the possible variation in fibril density across tissue thickness, and therefore the information obtained on fibril content and angular orientation at any point on the ocular surface, was assumed to remain depth-independent. Additionally, the quantification process was conducted based on annular areas across the full eye and resulted in the preferential fibrils becoming slightly lower in some locations. For instance, while the cornea diameters (limbus-to-limbus) were slightly different in the temporal-nasal and superior-inferior directions, this could not be considered in the analysis and as a result, the data points representing the limbus were not exactly located along the annulus [45].

This study has attempted to overcome the challenges caused by the preparation method using an effective process, through which the X-ray data is shifted to locations that closely represent their positions on the eye’s 3D surface. Zernike polynomials were then used to mathematically describe the X-ray data in their new locations and to present them in a form that enables their direct use in building numerical models of the ocular globe. In the absence of developments that would enable ocular globes to be scanned while intact, the process offers a quick, reliable and repeatable method to use fibril distribution data covering the whole eye surface.

In the central cornea (zone ‘a’), the more regular fibril distribution meant that Zernike fitting was more accurate compared with the other zones including the limbus, equatorial sclera and posterior sclera. Care is further needed when choosing the order of Zernike polynomials as low orders can lead to excessive smoothening of the data, while high orders can lead to overfitting and fluctuations in predicted data. Order 10 was found to be a suitable choice considering the data that were used in this study from seven human eyes (Fig 4). This is because Zernike polynomials appear to have been successful in removing the extreme fluctuations in data while avoiding overfitting.

In conclusion, the study presents an important step in the construction of numerical models of the ocular globe where the regional and anisotropic variation in stiffness of tissue are controlled based on actual fibril distribution maps obtained from X-ray scanning. It introduces a method to overcome imperfections in the data resulting from the method used to prepare the tissue for X-ray analysis. The data used in this research belonged to 7 ex-vivo, healthy human eyes but the method used to analyse the results are equally suitable for eyes with ectasia or surgeries.

## Supporting information

S1 File Raw X-ray data before and after reversing the dissected geometry. (ZIP)
